# Economics of immunization information systems in the United States: assessing costs and efficiency

**DOI:** 10.1186/1478-7547-4-15

**Published:** 2006-08-22

**Authors:** Diana L Bartlett, Noelle-Angelique M Molinari, Ismael R Ortega-Sanchez, Gary A Urquhart

**Affiliations:** 1 Immunization Services Division, National Center for Immunization and Respiratory Diseases, Centers for Disease Control and Prevention, 1600 Clifton Rd., NE, MS-E62 Atlanta, GA, 30333, USA; 2 Viral Diseases Division, National Center for Immunization and Respiratory Diseases, Centers for Disease Control and Prevention, 1600 Clifton Rd., NE, MS-A47, Atlanta, GA, 30333, USA

## Abstract

**Background:**

One of the United States' national health objectives for 2010 is that 95% of children aged <6 years participate in fully operational population-based immunization information systems (IIS). Despite important progress, child participation in most IIS has increased slowly, in part due to limited economic knowledge about IIS operations. Should IIS need further improvement, characterizing costs and identifying factors that affect IIS efficiency become crucial.

**Methods:**

Data were collected from a national sampling frame of the 56 states/cities that received federal immunization grants under U.S. Public Health Service Act 317b and completed the federal 1999 Immunization Registry Annual Report. The sampling frame was stratified by IIS functional status, children's enrollment in the IIS, and whether the IIS had been developed as an independent system or was integrated into a larger system. These sites self-reported IIS developmental and operational program costs for calendar years 1998–2002 using a standardized data collection tool and underwent on-site interviews to verify reported data with information from the state/city financial management system and other financial records. A parametric cost-per-patient-record (CPR) model was estimated. The model assessed the impact of labor and non-labor resources used in development and operations tasks, as well as the impact of information technology, local providers' participation and compliance with federal IIS performance standards (e.g., ensuring the confidentiality and security of information, ensure timely vaccination data at the time of patient encounter, and produce official immunization records). Given the number of records minimizing CPR, the additional amount of resources needed to meet national health goals for the year 2010 was also calculated.

**Results:**

Estimated CPR was as high as $10.30 and as low as $0.09 in operating IIS. About 20% of IIS had between 2.9 to 3.2 million records and showed CPR estimates of $0.09. Overall, CPR was highly sensitive to local providers' participation. To achieve the 2010 goals, additional aggregated costs were estimated to be $75.6 million nationwide.

**Conclusion:**

Efficiently increasing the number of records in IIS would require additional resources and careful consideration of various strategies to minimize CPR, such as boosting providers' participation.

## Background

One United States national health objective for 2010 is to have at least 95% of U.S. children aged <6 years participate in fully operational population-based immunization registries or immunization information systems (IIS) [1]. To achieve this objective the Centers for Disease Control and Prevention (CDC), through its National Immunization Program (NIP) awarded $262 million in immunization grants from 1994 to 2002 to develop and operate IIS in the 50 states, the District of Columbia, five urban areas, and all U.S. territories (CDC/Kim Lane, unpublished data, 2003). During this time period, the National Vaccine Advisory Committee recommended that the CDC determine the resources needed to further develop and maintain these systems as part of the requirements to ensure sustainable IIS funding [2].

As of 2002, approximately 43% of children of U.S. children <6 years of age had two or more vaccinations recorded in an IIS [3]. Although important progress has been made in populating these systems with vaccination records, achieving the 2010 IIS objective would not only require additional resources or funds but also a better understanding of how these resources can effectively be allocated to each IIS. Therefore, characterizing IIS development and operational costs as well as identifying other factors that affect IIS efficiency could assist public health agencies in planning, strengthening and further developing these systems.

Using a nationally representative sample of IIS, this article evaluates economic determinants of IIS performance by estimating the influence of development and management costs of in-house and contracted labor and non-labor and resources used by IIS centralized systems. It also measures the effect of standards and other factors affecting the functioning of such systems, determines the threshold of patient records needed to minimize average cost per patient record (CPR), and identifies strategies to increase efficiency.

## Methods

### Sample and setting

Data were collected from a national sampling frame of the 56 states/cities that received federal immunization grants under Public Health Service Act §317b and completed the CDC/NIP 1999 Immunization Registry Annual Report – a self-administered questionnaire applied to immunization program managers. This questionnaire assessed the percentage of children <6 years of age with >2 immunizations enrolled in an IIS, the percentage of provider sites participating in the IIS, whether the IIS operated independently or integrated with other health information systems, and evaluated the functional status of the IIS according to 12 federal IIS performance standards (e.g., ensuring the confidentiality and security of information, ensure timely vaccination data at the time of patient encounter, produce official immunization records) defined in more detail elsewhere [3].

Table 1 describes the IIS in the sampling frame. The sampling frame used the measures derived from the 1999 annual report to stratify IIS by how well they functioned (a score based on the number of federal IIS functional standards achieved), the percentage of children <6 years of age enrolled in the IIS, and whether the IIS had been developed as an independent system or was integrated into a larger public health system.

**Table 1:** Number of immunization information systems (IIS) stratified* by child enrollment and IIS functional score

		**Score of achievement of IIS functional standards^†^**		
		
		<37.1%	≥37.1% & <69.9%	69.9% +
		Low^§^	Medium	High
IIS integrated within larger system				
Children <6 years of age enrolled				
<1.7%	Low	Unknown	0	4 (3)
≥1.7% & <53.5%	Medium	0	5 (2)	5 (2)
53.5% +	High	0	7 (2)	1 (1)
IIS operating as independent system				
Children <6 years of age enrolled				
<1.7%	Low	Unknown	0	2 (2)
≥1.7% & <53.5%	Medium	0	4 (2)	4 (2)
53.5% +	High	0	4 (2)	3 (2)

		17 (4)	20 (8)	19 (12)

* Two IIS were randomly sampled from each stratum or all IIS sampled if ≤ 2 in a stratum with the exception of high functional score and low child enrollment where 3 were sampled because 2 states shared the same IIS. Number sampled is in parentheses. Cut points in the strata were chosen to represent natural breaks in the data.

^† ^Indicates an IIS minimum functional standard recommended by the National Center for Immunization and Respiratory Diseases (NCIRD) Technical Working Group. .

^§ ^17 IIS were under development and fell into a stratum of <37.1% functional score and <1.7% child enrollment; 4 IIS were randomly sampled from this group for participation in this study.

IIS were classified as 'low', 'medium', or 'high' in terms of children enrolled if the percentage of children enrolled was < 1.7%, >1.7% and < 53.5%, or > 53.5%. IIS were classified as 'low', 'medium', or 'high' in terms of IIS functionality score if the percentage of IIS functional standards achieved was < 37.1%, >37.1% and < 69.9%, or > 69.9%. These cut points were chosen to represent natural breaks in the data. Two IIS were randomly sampled from each stratum or all IIS sampled if ≤ 2 in a stratum with the exception of high functional score and low child enrollment where 3 were sampled because 2 states shared the same IIS. 17 IIS were under development and fell into a stratum of <37.1% functional score and <1.7% child enrollment; 4 IIS were randomly sampled from this group for participation in this study. The final sample consisted of 24 IIS. These IIS varied by duration of operation (about half began IIS development in 1993) and targeted age range (most IIS collected vaccination data on persons aged 0–18 years old).

### Data collection

In August 1999, the IIS sites in the sample collected developmental and operational program costs for calendar years 1998 and 1999. From May to October 2003, researchers visited the selected sites with on-site interviews to collect IIS expenditures for calendar years 2000–2002 and verify data collected previously. Since the 1999 assessment, many IIS had upgraded to web-based systems, expanded their partnerships and marketing efforts to the private sector, and found additional funding sources. These data were combined with data from CY 1998–1999 to allow analysis over a five year period. All sites reported data from their financial management offices and internal records.

Annual expenditures were collected as line items for in-house personnel (including fringe benefits), contractor full-time equivalents (FTEs), equipment, supplies, travel, contractual expenses, other expenses, and indirect costs. Object class definitions developed by the Office of Management and Budget (OMB) for Federal program costs were used to categorize expenditures [4].

Data on IIS expenditures by source of funds were also collected. These included federal immunization grants under Public Health Service Act §317b; 'other' federal sources such as other CDC funds, Maternal and Child Health block grants, Vaccines for Children program funds, or funds from the Centers for Medicare and Medicaid Services; state and local government funds; private sector funds; in-kind contributions; and funds from other sources, such as fees from IIS subscribers. Personnel and contractual cost line items included costs to develop and operate the IIS, to enter data at the central IIS, to evaluate the IIS, and for marketing efforts to recruit parents and health providers to participate in the IIS. Only costs to operate the central IIS within a geographic area were included (i.e., costs for daily operations of local IIS within a state were excluded). IIS costs to providers were excluded. For IIS that were a part of a larger, integrated information system (i.e., system includes lead and metabolic screening data), only the central IIS system's portion of these costs were collected, not the costs to develop and operate the entire integrated system.

All IIS line item costs were separated into developmental and operational costs. Developmental costs were defined as costs incurred prior to any immunization data submission, storage, or access by an immunization provider. Software and hardware upgrade costs were also considered developmental costs, especially in regard to upgrading to a web-based system. Operational costs were defined as costs to maintain the current status of the IIS after immunization providers began to submit data or access IIS records.

### Economic analysis

Annual mean IIS costs were estimated for labor, non-labor, development and operations by funding source and by number of patient records (all ages) in the IIS. Labor costs included personnel salaries and fringe benefits of persons who were city/state government employees as well as contractor full-time equivalents (FTEs) who worked as IIS employees. All other costs, including indirect costs, were included in non-labor costs. Cost data from 1998–2001 were adjusted to 2002 dollars using the Consumer Price Index [5]. Descriptive statistics were weighted to be nationally representative and analyzed using NLOGIT econometric software version 3.0.2.

To examine factors that influenced average CPR in IIS and estimate prospective costs of achieving the Healthy People 2010 goals, a behavioral cost equation was estimated using weighted least squares with site-specific fixed effects. By allowing for site-specific heterogeneity, one could capture the effect on CPR of any unobserved site-specific characteristics. This greatly improved the precision of estimates. The equation for the CPR of site *i*'s IIS in period *t *was represented by:

ln⁡(AC)it=αi+βξξit+βwln⁡wit+βkln⁡rkit+βQ1Qit+βQ2Qit2+βXXit+eit     (a)
 MathType@MTEF@5@5@+=feaafiart1ev1aaatCvAUfKttLearuWrP9MDH5MBPbIqV92AaeXatLxBI9gBaebbnrfifHhDYfgasaacH8akY=wiFfYdH8Gipec8Eeeu0xXdbba9frFj0=OqFfea0dXdd9vqai=hGuQ8kuc9pgc9s8qqaq=dirpe0xb9q8qiLsFr0=vr0=vr0dc8meaabaqaciaacaGaaeqabaqabeGadaaakeaacyGGSbaBcqGGUbGBcqGGOaakcqWGbbqqcqWGdbWqcqGGPaqkdaWgaaWcbaGaemyAaKMaemiDaqhabeaakiabg2da9GGaciab=f7aHnaaBaaaleaacqWGPbqAaeqaaOGaey4kaSIae8NSdi2aaSbaaSqaaiab=57a4bqabaGccqWF+oaEdaWgaaWcbaGaemyAaKMaemiDaqhabeaakiabgUcaRiab=j7aInaaBaaaleaacqWG3bWDaeqaaOGagiiBaWMaeiOBa4Maem4DaC3aaSbaaSqaaiabdMgaPjabdsha0bqabaGccqGHRaWkcqWFYoGydaWgaaWcbaGaem4AaSgabeaakiGbcYgaSjabc6gaUjabdkhaYjabdUgaRnaaBaaaleaacqWGPbqAcqWG0baDaeqaaOGaey4kaSIae8NSdi2aaSbaaSqaaiabdgfarjabigdaXaqabaGccqWGrbqudaWgaaWcbaGaemyAaKMaemiDaqhabeaakiabgUcaRiab=j7aInaaBaaaleaacqWGrbqucqaIYaGmaeqaaOGaemyuae1aa0baaSqaaiabdMgaPjabdsha0bqaaiabikdaYaaakiabgUcaRiab=j7aInaaBaaaleaacqWGybawaeqaaOGaemiwaG1aaSbaaSqaaiabdMgaPjabdsha0bqabaGccqGHRaWkcqWGLbqzdaWgaaWcbaGaemyAaKMaemiDaqhabeaakiaaxMaacaWLjaWaaeWaceaacqqGHbqyaiaawIcacaGLPaaaaaa@8024@

where *AC*_*it *_is average cost for site *i *in period *t*, *α*_*i *_is the site-specific fixed effect for site *i*, *w*_it _is the wage of labor, *rk*_*it *_is expenditures on capital, *Q*_*it *_is total patient records in IIS, ξ_*it *_is achievement of IIS functional standards, and the *X*_*it *_are site characteristics. The regression error for site *i *in period *t *is *e*_*it*_. Each of the 24 sampled IIS were observed once per year for a period of five years. Therefore, the sample consisted of 120 data entries. In the final sample, each entry referred to the observation of a specific IIS in a given year, henceforth an IIS-year.

Average CPR was transformed by the natural log as were wages and expenditures to better approximate their relationship to average cost, which was log-normally distributed. In order to allow for the presumed U-shape of the average cost curve, output was specified in quadratic form. This fixed effects model was estimated using weighted least squares to account for stratified sampling with robust estimation of the covariance matrix. Standard errors were corrected using the finite population correction factor, since sample size approached population size, *n *→ *N*.

The cost of achieving the Healthy People 2010 goal for each sampled IIS was calculated using parameters retrieved from the average cost estimation, discounting at a rate of 3%. For simplicity, it was assumed that each IIS met all 12 functional standards (the Healthy People 2010 IIS goal includes having 95% child participation in an IIS that is fully functional, i.e., meets the 12 IIS functional standards) while other variables were evaluated at their 2002 values. It was assumed, again for simplicity, that the gap between current child participation and 95% child participation was closed at a linear rate of one-eighth the gap per year, while the IIS continued to maintain the average child population growth rate that it had exhibited in the previous five years. IIS that had already achieved or exceeded 95% child participation were assumed to continue to maintain current participation levels by growing with the child population.

In order to illustrate the importance of vaccine provider participation in an IIS, the impact of simultaneously achieving 95% child participation and 95% provider site participation was also examined. The same assumptions used previously were employed. In addition to child participation, the gap between current provider site participation and 95% provider site participation would close at a linear rate of one-eighth the gap per year. Sampled IIS totals were weighted and the national cost for all 56 IIS to achieve the Healthy People 2010 goal was calculated with and without 95% provider site participation.

## Results

Table 2 presents summary statistics for estimated annual labor, non-labor, and total costs by category of spending, development or operations, and IIS size in terms of patient records in the IIS database. The estimated mean annual immunization IIS total cost ranged from $919,421 for IIS with 860,000 patient records or less to $1,487,324 for IIS with over 3.4 million patient records. Estimated mean annual labor costs increased as patient records in an IIS increased from $359,381 for IIS with <860,000 patient records to $1.1 million for IIS with the most patient records. Estimated mean annual non-labor costs were a larger proportion of estimated mean annual total costs for IIS with <3.4 million patient records. Estimated mean annual development costs were greatest for IIS with <860,000 patient records while IIS with 1.7 million-3.4 million patient records had the highest estimated mean annual operations costs.

**Table 2:** Estimated annual immunization information system (IIS) costs^† ^by category of spending and IIS size^§^

		**IIS size in thousands of patient records**				
		
		**0 to 860**	**860 to 1,720**	**1,720 to 2,580**	**2,580 to 3,440**	**Over 3,440**
IIS years*	**N**	79	12	17	10	2
	**%**	66	10	14	8	2
						
**Total Population**	**Mean**	3,606,105	7,494,123	5,960,495	10,533,124	21,538,762
	(StDev)	(3,150,708)	(2,363,222)	(4,422,892)	(5,424,221)	(280,246)
						
**Child Population**	**Mean**	298,844	654,015	507,237	911,436	2,074,273
	(StDev)	(260,203)	(178,398)	(429,933)	(564,371)	(49,338)
						
**Child Participation**	**Mean**	29%	63%	72%	59%	44%
	(StDev)	(0.54)	(0.43)	(0.29)	(0.26)	(0.14)
						
**Non Labor Cost**						
Development	**Mean**	285,509	142,031	163,740	291,374	0
	(StDev)	(578,487)	(286,985)	(337,863)	(481,137)	0
Operations	**Mean**	274,531	696,142	596,064	679,860	454,966
	(StDev)	(769,412)	(541,044)	(923,378)	(1,029,998)	(124,004)
Total Non labor	**Mean**	560,040	838,174	759,804	971,233	366,191
	(StDev)	(1,039,098)	(629,139)	(1,098,955)	(1,192,329)	(301,553)
						
**Labor Costs**						
Development	**Mean**	248,554	100,941	63,814	131,161	102,039
	(StDev)	(623,388)	(159,685)	(135,881)	(370,508)	(204,079)
Operations	**Mean**	110,827	419,317	554,676	480,370	1,019,093
	(StDev)	(219,691)	(559,807)	(337,150)	(534,342)	(618,350)
Total Labor	**Mean**	359,381	520,258	618,490	611,531	1,121,132
	(StDev)	(600,991)	(489,775)	(304,919)	(601,790)	(414,271)
						
**Overall Costs**						
**Development**	**Mean**	534,063	242,972	227,554	422,534	102,039
	(StDev)	(813,133)	(394,978)	(379,468)	(700,865)	(204,079)
**Operations**	**Mean**	385,358	1,115,459	1,150,740	1,160,230	1,019,093
	(StDev)	(850,668)	(914,221)	(1,091,372)	(1,234,443)	(618,350)
**Total**	**Mean**	919,421	1,358,431	1,378,294	1,582,764	1,487,324
	(StDev)	(1,121,652)	(900,971)	(1,237,504)	(1,592,114)	(715,824)

*IIS years connotes an observation for each of the 24 sampled IIS once per year over a five year period; therefore the sample for our analysis consisted of 120 data entries. Each entry refers to the particular observation of a specific IIS in a given year, henceforth an IIS-year

^† ^Dollars per IIS year inflated to 2002 dollars

^§ ^Cut points are based on 5 equal intervals between 0 and 3.6 million patient records

**Figure 1:**
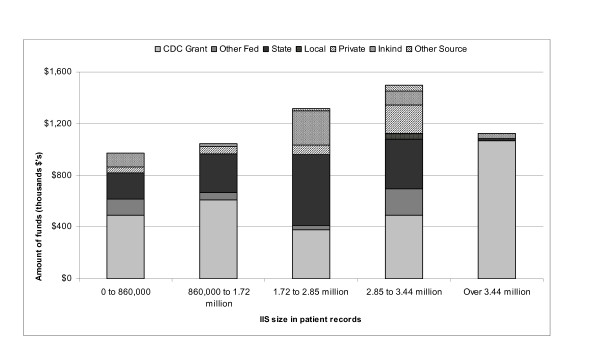
Average amount of annual expenditures^*^ by funding source^†^ and immunization information system size^§^, 1998-2002.^*^Dollars inflated to 2002 dollars.   ^†^Sources of funds included immunization grants from the Centers for Disease Control and Prevention (CDC), funding support from other CDC offices and other federal agencies, state government funds, local government funds, private donations, in-kind support, and other sources of funds (e.g., user fees).   ^§^Cut points in immunization information system size are based on five equal intervals between 0 and 3.4 million records

Figure 1 presents the distribution of funds by source and IIS size. In general, CDC immunization grants and state funds accounted for the majority of funding and increased with size of IIS. For IIS with less than 860,000 records, 64% of funds came from federal sources; 51% from CDC immunization grants and 13% from other federal sources. State funds accounted for 21% of the total. For IIS with between 860,000 and 1.72 million records, federal funds accounted for 64% of total funds with 59% from CDC 317 immunization grants. State governments contributed 29% of total funds. IIS with patient records between 1.72 and 2.85 million experienced a somewhat different mix of funding sources. Federal funds accounted for 31% of the total with CDC immunization grants representing 29% and other federal funds comprising 2%. State funding contributed 42% of the total. IIS with 2.85 to 3.44 million records received 47% of total funds from federal sources with 33% from CDC immunization grants. These IIS received 26% and 3% of funds from state and local governments respectively. IIS with more than 3.44 million records received 95% of funds from CDC immunization grants. The remaining 5% was composed of 4% in-kind contributions and 1% state government contributions.

Table 3 presents results from the fixed effects, weighted least squares estimation of the average cost function for sites with patient records > 0. Site-specific effects were strongly significant, indicating that unique characteristics of each project site did influence CPR. Table 3 presents statistically significant coefficients and the impact of associated variables on average cost. The model did not directly compare integrated with independent IIS in the regression due to collinearity. The impact of independent variables on average costs are displayed as marginal effects or elasticities impacts in the table. Marginal effects are displayed for categorical variables indicating the impact of the IIS having the associated characteristic in a given year. Elasticities, which are sensitivity measures of change, are estimated for continuous variables used in the regression. Specifically, other things remaining equal, the elasticity measures the proportional or relative response of average cost given a one percent increase in a specific independent variable (e.g., personnel wages or government funds).

**Table 3:** Factors influencing average costs per patient record using a fixed effects weighted least squares model

**Variable**	**Coefficient**	**Impact**
Immunization information system (IIS) record created within 6 weeks of birth ^¶^	0.198356 **	$0.22^†^
Clinic access to IIS data during patient encounter ^¶^	-0.370097***	-$0.31^†^
Update record in IIS within 30 days of vaccine administration ^¶^	0.342897***	$0.41^†^
Identify persons needing vaccine reminder/recall notifications ^¶^	-0.179461**	-$0.16^†^
Automatically produce immunization coverage reports ^¶^	-0.117172*	-$0.11^†^
Produce official immunization records ^¶^	-0.190538**	-$0.17^†^
Promote IIS data accuracy and completeness ^¶^	0.411732***	$0.51^†^
Received other federal government funding	0.328083***	$0.39^†^
Received state government funds	-0.535686***	-$0.41^†^
Received local government funds	0.716021*	$1.05^†^
Received private organization funds	-0.272138**	-$0.24^†^
Received in-kind support	0.579388***	$0.78^†^
Proportion of vaccine providers participating in the IIS	1.364995***	0.25
(Proportion of vaccine providers participating in the IIS)^2^	-0.921197*	
Proportion of IIS patient records that are children	-1.523795***	-0.71
Number of patient records in the IIS (all ages)	-0.000003***	-1.96
(Number of patient records in the IIS)^2^	0.000000***	
Average wage of development personnel^§^	-0.047884***	-0.05
Average wage of operations personnel^§^	-0.122950***	-0.12
Average wage of development contractor^§^	-0.019642***	-0.02
Spending on development equipment^§^	0.062248***	0.06
Spending on operations equipment^§^	0.027393***	0.03
Spending on development supplies^§^	0.072092***	0.07
Spending on operations travel^§^	0.049979***	0.05
Other spending on development^§^	0.050567***	0.05
Contractual spending on development^§^	0.036724***	0.04
Contractual spending on operations^§^	0.053036***	0.05

Dependent variable is natural log of Avg Cost | Only significant variables are displayed | Variables not displayed include: population density, standards 1,5–8, wage of operations contractor, supply, indirect & other expenses for operations, travel & indirect expenses for development, lagged deviation between funds & expenses, and receipt of CDC grant & other funds | Adj R^2 ^= 0.96, SSR = 1.02, lnL = 62.19, N = 91 | *, **, *** indicate statistical significance at p < 0.1, p < 0.05, p < 0.01 respectively |^2 ^indicates squared variable.

† indicates marginal effect instead of elasticity.

^§ ^indicates natural log.

^¶ ^indicates IIS minimum functional standard recommended by the National Center for Immunization and Respiratory Diseases (NCIRD)'s Technical Working Group –

Funding sources affected average costs although receipt of CDC immunization grant funds did not significantly affect average costs. Receipt of other federal, local, and in-kind funds tended to be associated with increased average costs. Specifically, receipt of local government funds and in-kind contributions was associated with an increased CPR by $1.05 and $0.78 respectively while receipt of other Federal funds was associated with an increased CPR by $0.39. Conversely, receipt of state or private funds was associated with a reduced CPR by $0.41 and $0.24 respectively. Funds from other sources did not significantly affect average costs.

Achievement of the 12 IIS functional standards also impacted the average CPR, though certain standards impacted more than others. Specifically, ensuring data accuracy and completeness increased average cost by $0.51 per record while IIS ability to receive and process vaccination data within one month of vaccine administration increased CPR by $0.41. These were followed in impact by the ability for a clinic to access IIS data during patient visits (-$0.31) and the ability to create an IIS record within six weeks of birth ($0.22). Average costs for IIS that met all 12 functional standards increased by $0.21 per record compared to IIS that did not meet all standards. IIS ability to identify persons needing vaccine reminder/recall notifications decreased the CPR by $0.16, and the ability to automatically produce immunization coverage reports and official immunization records decreased average costs by $0.11 and $0.17 respectively.

Population characteristics and demographics are usually important determinants of average cost. While population density did not significantly influence CPR, as the proportion of IIS patient records belonging to children increased, average costs declined. A 1% increase in the proportion of child records was associated with 0.71 percent decline in average cost. This effect was both strong and highly significant.

A 1% increase in the wages of development personnel and contractors was associated with declines in average cost of 0.05% and 0.02% respectively. Similarly, the elasticity of operations personnel wages with respect to average cost was – 0.12. Operations contractor wages did not significantly affect average cost. Expenditures on operating supplies, other purchases, and indirect costs (i.e., overhead) did not significantly affect average costs; nor did expenditures on travel or indirect overhead for development. For the remaining factors, the effect was positive and significant but uniformly inelastic.

The results for patient records displayed in Table 2 make a strong case for economies of scale. As number of records increased, average estimated costs declined at an increasing rate, displaying the expected U-shape. For the average IIS, a 1% increase in patient records resulted in 1.96% decline in CPR. Clearly the average registry, with 980,000 records, was still experiencing declining average costs and had not yet achieved minimum efficient scale.

**Figure 2:**
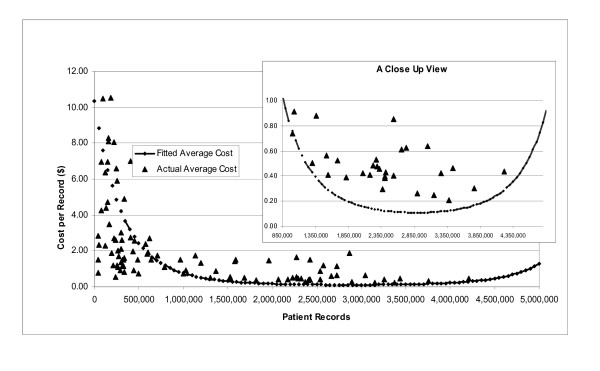
The influence of immunization information system size on cost* per patient record. * Cost per record inflated to 2002 dollars.

Figure 2 displays the relationship between average cost and patient records given technology, etc. Average cost declined sharply over the first one million records and then began to slow. Minimum average cost was $0.09 per patient record with efficient scale ranging from 2.9 million records through 3.2 million records, at which point average cost began to rise again. Note that as of 2002, only 20% of IIS were near efficient scale.

**Figure 3:**
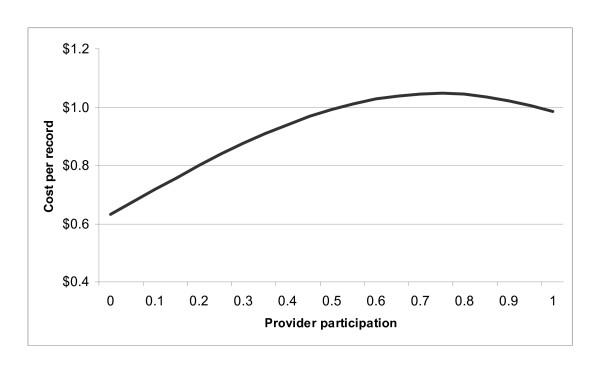
The influence of vaccine provider participation* in immunization information system costs^† ^per patient record. * Public and private vaccine service delivery locations that maintain permanent vaccination records and submit vaccination data to an immunization information system routinely (i.e., within the last six months of a calendar year) ^†^ Cost per record inflated to 2002 dollars

Increased provider participation was associated with increased average costs up to a point, after which costs declined. Figure 3 displays the relationship between provider site participation and CPR. At the current average provider participation rate of 32%, a 1% increase in provider participation was associated with a 0.25% increase in CPR. As provider participation increased, however, the association became smaller. Levels above 80% participation were associated with a decline in average costs.

Results from these calculations indicated a cost of $75.6 million ($76.9 million with no discounting) in 2002 dollars over eight years for all 56 IIS to meet the Healthy People 2010 goal, assuming that all other parameters remain the same. The cost to achieve the Healthy People 2010 goal as well as 95% provider participation amounted to $56.4 million ($57.7 million with no discounting) in 2002 dollars for all 56 IIS. Note that this figure only includes the cost to the central IIS and does not account for costs to providers.

## Discussion

This study was designed to characterize the average costs of IIS and to estimate costs for IIS nationwide to be fully functional, population-based, and contain >95% of the children <6 years of age in their geographic areas. States developing IIS can estimate the costs in their plans to develop and operate an IIS based on their population size and anticipated IIS size. Based on results from the average cost model, it costs about $0.20 per patient record to achieve full functional status measured by the 12 IIS functional standards.

In general, operations expenses tended to increase with IIS size while development expenses declined. This appeared to hold true within categories as well. An increase in expenditures was associated with an increase in IIS size, but IIS at or near efficient size had more diverse sources of funds. Funding sources might affect the operation of IIS but the effect on average costs is not highly significant.

Past studies collected costs through 1997–1998, when many IIS had not yet migrated to web-based systems [2,6-10]. These studies also focused on relatively young IIS. Modern IIS systems have more demands on their resources than their predecessors. IIS are directed now to develop added capabilities such as vaccine management, adverse event reporting, lifespan vaccination histories, and linkages with electronic data sources to encourage provider participation. As a public health electronic system, their concerns would also focus on how they will fit into the President's proposed National Health Information Network and compete for federal resources [11].

While the literature lacks examples of IIS costs at the national level, it also lacks studies that characterize the variety of funding sources used in IIS development and operations and the impact of economies of scale on CPR. Having the most recent data available, including funding sources, may account for the technological changes and new demands placed on the modern IIS. Timely cost data and investigating economies of scale are essential for planning national IIS goals through 2010.

Provider participation is also essential for IIS to achieve their Healthy People 2010 goal. Up front investments by the IIS to integrate with electronic health records, patient management systems, and billing systems from the provider office could encourage provider participation by reducing data entry costs to the provider. Some of the IIS systems sampled devoted resources to provide computers and data entry services to providers and to integrating their system with other public health systems. These costs were included in the central IIS development and operations costs. While costly at first, the marginal cost of provider participation tended to decline as provider participation increased. As IIS size increased, CPR declined.

This study is subject to several limitations. First, time has not been modeled explicitly in this analysis. The results assume a one time, lump-sum cost of achieving the functional standards and that the gap between 2002 child participation and 95% child participation will be closed linearly. In reality, it is likely that IIS will achieve functional standards over time and child participation may increase nonlinearly over time. The extent that actual IIS achievements of functional standards differ from our assumptions may affect the cost of those achievements. Although it is not clear whether the effect would be positive or negative, in general, technological improvements tend to lead to cost savings. Therefore, to the extent that achievement of functionality represents technology improvement, fully functional IIS should operate more efficiently than their less functional counterparts. Second, the data were self-reported using data from IIS financial systems and internal records. The quality of available data and the completeness of the state financial records varied between the sites. While estimates are comprehensive in terms of types of costs considered and in-person interviews were conducted, it is possible that some expenditures may not have been captured.

Third, this is a study of costs incurred by the central IIS of federally funded IIS grantees over a five-year period. Resources did not permit the formal collection of costs incurred by provider practices or the costs incurred for daily operations of local IIS that export data to the central IIS. While all of the state IIS in the sample included costs of contracts with local health districts to obtain local vaccination data, these contracts did not fully support local IIS operations. Regarding costs to providers, costs for data entry were collected if the central IIS paid for these services. Two recent studies of IIS costs included the costs to providers to enter data and use the IIS for immunization assessment, immunization history reports, and other reports, but only included a small number of private practices in a small geographic area [9,12]; we could not generalize these costs to our national model. Costs to collect this data for the 24 sampled sites were prohibitive. Fourth, depreciation was not accounted for, nor were external factors such as managed care penetration, the number of public or private providers, etc. Nevertheless, this is the first study to estimate the funding needed to support IIS based on a true national sample.

While further analysis is necessary to better understand development and operations of IIS, some implications for policy are clear. Increases in the scale of operations are necessary in order to take advantage of substantial economies of scale. While minimum efficient scale may not be a feasible achievement for IIS with smaller populations, any increase in scale up to 2,900,000 records should result in declining average cost and greater efficiency. IIS with smaller populations may consider partnering with other regions and states to improve their economies of scale. IIS policy makers should also consider improving functionality and the adoption of new information technology; this may lead to efficiency improvements that can reduce total costs. IIS are also encouraged to seek out diverse resources for long-term sustainability. Funding sources vary in accountability requirements and IIS with more diverse funding sources may face greater accountability resulting in more efficient practices. Finally, IIS should enhance electronic linkages with providers to encourage provider participation. Provider costs to use an IIS and/or submit data to an IIS can deter participation and it would be well worth the investment of IIS funds to assess the costs to participating providers and implement strategies to decrease these costs. Obtaining data electronically from electronic health records, managed care organizations, and other large vaccination data sources, could decrease CPR as well as the costs to providers to submit data. Also, for states facing hiring freezes or difficulties in recruiting technical staff, hiring contractors may be a cost-effective option; developmental contractor salaries were associated with a slight decrease in average costs and operational contractor wages did not significantly affect average costs.

## Conclusion

Although complementary resources and efforts will be needed from IIS nationwide, achievement of the 2010 national IIS objective is possible. Efficiently increasing the number of children in IIS, however, would not only require the identification of additional and sustainable resources but, for the most part, a careful consideration of various strategies that minimize IIS' average costs. Among such strategies, substantial increments in providers' participation could significantly decrease CPR.

## Abbreviations

CDC-Centers for Disease Control and Prevention

CPR-Cost per patient record

FTE-Full time equivalents

IIS-Immunization information systems

NIP-National Immunization Program (now the National Center for Immunization and Respiratory Diseases)

OMB-Office of Management and Budget

US-United States

## Competing interests

The author(s) declare that they have no competing interests.

## Authors' contributions

DLB was responsible for instrument development, data collection, and manuscript preparation. N-AMM analyzed the data, interpreted the results and participated in manuscript preparation. IRO-S participated in data analysis and interpretation of results and manuscript preparation. GAU participated in the overall study design, instrument development, and data collection. All authors read and approved the final manuscript.
